# Overview of Evidence-Based Chemotherapy for Oral Cancer: Focus on Drug Resistance Related to the Epithelial-Mesenchymal Transition

**DOI:** 10.3390/biom11060893

**Published:** 2021-06-16

**Authors:** Jingjing Sha, Yunpeng Bai, Huy Xuan Ngo, Tatsuo Okui, Takahiro Kanno

**Affiliations:** Department of Oral and Maxillofacial Surgery, Shimane University Faculty of Medicine, 89-1 Enya-Cho, Izumo, Shimane 693-8501, Japan; jsswjbnjw@gmail.com (J.S.); xyywq@126.com (Y.B.); ngoxuanhuy158@gmail.com (H.X.N.); tokui@med.shimane-u.ac.jp (T.O.)

**Keywords:** EMT, chemotherapy, chemoresistance, oral squamous cell carcinoma

## Abstract

The increasing incidence of resistance to chemotherapeutic agents has become a major issue in the treatment of oral cancer (OC). Epithelial-mesenchymal transition (EMT) has attracted a great deal of attention in recent years with regard to its relation to the mechanism of chemotherapy drug resistance. EMT-activating transcription factors (EMT-ATFs), such as Snail, TWIST, and ZEB, can activate several different molecular pathways, e.g., PI3K/AKT, NF-κB, and TGF-β. In contrast, the activated oncological signal pathways provide reciprocal feedback that affects the expression of EMT-ATFs, resulting in a peritumoral extracellular environment conducive to cancer cell survival and evasion of the immune system, leading to resistance to multiple chemotherapeutic agents. We present an overview of evidence-based chemotherapy for OC treatment based on the National Comprehensive Cancer Network (NCCN) Chemotherapy Order Templates. We focus on the molecular pathways involved in drug resistance related to the EMT and highlight the signal pathways and transcription factors that may be important for EMT-regulated drug resistance. Rapid progress in antitumor regimens, together with the application of powerful techniques such as high-throughput screening and microRNA technology, will facilitate the development of therapeutic strategies to augment chemotherapy.

## 1. Introduction

As the second leading cause of death globally, cancer still represents a major public health challenge. The latest epidemiological analysis of the incidence of cancer in the USA indicated that 4950 people are diagnosed with cancer every day, with an annual incidence of 1,806,590 [1]. Although not common in developed countries, oral cancer (OC), a type of head and neck cancer (HNC), is still the sixth leading type of cancer in the world, with an estimated incidence of 275,000 cases per year [2]. Two-thirds of OC patients reside in developing countries. There is huge geographic variation in the incidence of OC, with an approximately 20-fold difference between the countries with the lowest and highest rates [3]. In high-risk countries, such as India and Sri Lanka, OC is the main cancer in men and accounts for up to 25% of all new cases of cancer in these countries. However, OC accounts for only 1–3% of all malignancies in countries with low incidence rates, such as the UK [4].

Martinez reported that recurrent and metastatic HNC, especially head and neck squamous cell carcinoma (HNSCC), are frequently seen in daily clinical practice. Between 20% and 40% of patients with stage I/II disease, and more than 70% of patients with stage III/IV disease at first diagnosis treated with curative intent, will show recurrence [5]. As the main pathological type of OC, oral squamous cell carcinoma (OSCC) accounts for at least 90% of all of these malignancies [6].

A report by the US Centers for Disease Control and Prevention (CDC) indicated that 36% of patients with OSCC have localized disease, while 43% have locoregional spread and 9% present with distant metastasis at the initial diagnosis [7]. Overall, locoregional recurrence is very common and leads to death in 40–60% of these patients, whereas less than 20% of patients die because of distant metastasis [5]. Frustratingly, the overall five-year survival rates for OC are around 50–60% even after decades of development of cancer treatments [8].

Regardless of diagnostic methods, numerous therapeutic strategies can be applied for OSCC treatment. Chemotherapy is the first-line treatment for various types of cancer, including OSCC [9]. However, the development of chemoresistance represents a challenge in chemotherapy [10]. Previous studies have shown that frequent application of high-dose chemotherapeutic agents has led to the emergence of chemoresistance, where overcoming this issue has become a major goal for researchers around the world.

Cancer cells have been shown to switch between molecular pathways and mechanisms to ensure their proliferation, invasiveness, and resistance to chemotherapeutic agents [11]. During this process, invading cancer cells acquire mesenchymal features, while losing cell polarity and intercellular tight junctions. This transition from epithelial to mesenchymal cells is designated as the epithelial-mesenchymal transition (EMT) [12]. The EMT was first identified in the 1970s as a feature of embryogenesis and wound healing, but its underlying mechanisms have since been studied extensively and used to explain carcinogenesis and tumor invasiveness [13]. The main characteristic of EMT during tumor metastasis is the loss of the adherent junction protein E-cadherin. A number of transcription factors participate in the regulation of E-cadherin, but only a few directly mediate its expression [14]. These EMT-activating transcription factors (EMT-ATFs), which include the Snail, TWIST, and ZEB families, bind specifically to the promoter of E-cadherin through E-boxes and inhibit its transcription [15], and thus play pivotal roles in the dynamic regulation of EMT, tumor metastasis, and resistance to chemotherapy agents [16].

Here, we refer to the National Comprehensive Cancer Network (NCCN) Chemotherapy Order Templates, especially with regard to OSCC/HNSCC [17]. We discuss the role of EMT in the emergence of resistance to the seven conventional chemotherapeutic agents shown in Table 1, which are the most widely used drugs for advanced and recurrent OSSC/HNSCC. This review provides valuable information regarding resistance to chemotherapy in OSCC and should therefore facilitate the development of solutions to this growing problem in cancer treatment.

## 2. Antimetabolites

### 2.1. Methotrexate

Methotrexate, formerly known as amethopterin, is an antifolate that belongs to a class of drugs known as antimetabolites [18]. As chemotherapy agents and immune suppressants, these drugs work by reducing or stopping the growth of cancer cells and inhibiting the immune system [19].

Administration of methotrexate as a single agent is the standard treatment for advanced, recurrent, and metastatic HNSCC [20,21]. Methotrexate is one of the most commonly used chemotherapeutic agents for palliative care in patients with recurrent HNSCC and shows response rate of 8–50%. This agent is also the standard used for comparison with many other drugs in phase III trials [22].

The target of methotrexate is dihydrofolate reductase (DHFR), an enzyme involved in tetrahydrofolate synthesis. The affinity of methotrexate to DHFR is about 1000 times greater than that of folate, allowing it to competitively inhibit the activity of the enzyme [23,24].

The conversion of dihydrofolate to the active tetrahydrofolate is catalyzed by DHFR. Folic acid is essential for biosynthesis of the pyrimidine nucleoside, thymidine, which is required for DNA synthesis [24]. Folate is also necessary for the synthesis of compounds based on purines and pyrimidines. Therefore, competitive inhibition of DHFR by methotrexate can suppress the synthesis of folate-based chemical compounds and inhibit the synthesis of DNA, RNA, thymidylates, and proteins [23].

**Figure 1:**
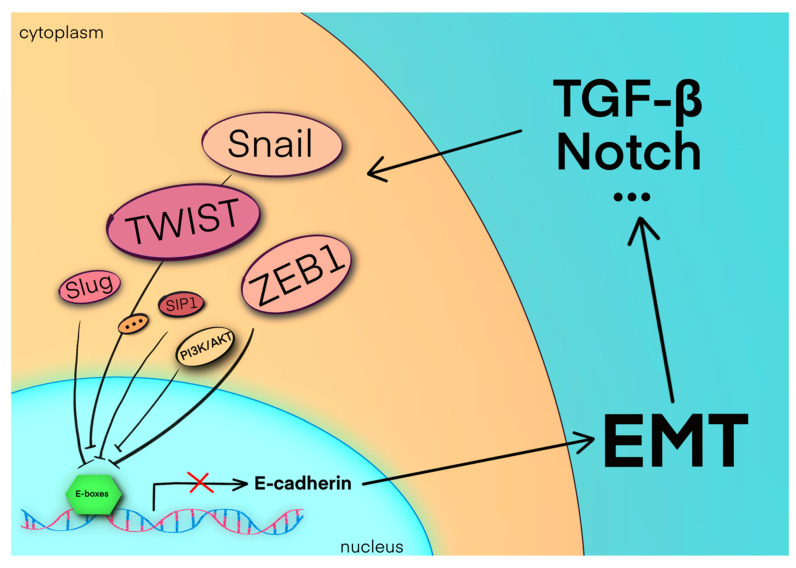
Binding of EMT-AFTs, such as Snail, TWIST, and ZEB1, to E-boxes in the promoter suppresses E-cadherin gene expression, which is the hallmark of EMT. The cells then acquire mesenchymal features and migration capability, and even the stem-cell-like features of the so-called cancer stem cells (CSCs). Moreover, several complex signaling pathways, such as the PI3K/AKT pathway, TGF-β pathway, Notch pathway, etc., participate in regulating the expression of EMT-AFTs, forming a feedforward loop that promotes the EMT process [26,27].

During the process of EMT, epithelial cells are transformed into cells with mesenchymal, migratory, and invasive characteristics, and these converted cells have the properties of stem cells [25]. The EMT-AFTs, such as Snail, TWIST, and ZEB1, work by binding to E-boxes in the promoter, thereby inhibiting the expression of E-cadherin [26]. Several complex signaling pathways, including TGF-β and PI3K/AKT, etc., participate in regulating the expression and activity of these transcription factors (Figure 1) [27].

In an in vitro study, methotrexate induced the expression of E-cadherin in the human-derived colon cell line, SW620, and the human-derived skin cell line, SK-MEL-28 [28]. Huang et al. [29] showed that methotrexate downregulated HDAC/EZH2, which is required for methylation of histone H3 on lysine 27, thereby leading to upregulation of E-cadherin. Activation of the EMT also leads to methotrexate resistance. Consistent with earlier molecular analyses, overexpression of Golgi membrane protein 1 (GOLM1) was shown to induce activation of the EMT pathway via upregulation of MMP13, eventually leading to drug resistance [30]. Upregulation of MMP13 protein expression is strongly correlated with lymph node metastasis in OSCC. High levels of MMP13 mRNA and protein expression were shown to be related to a poor prognosis of OSCC [31].

The overexpression of S-phase kinase-associated protein 2 (Skp2) is another factor closely associated with methotrexate resistance and EMT [32]. Ding et al. [33] reported that the expression of Skp2 was markedly elevated in methotrexate-resistant osteosarcoma (OS) cells. In addition, methotrexate-resistant OS cells with a stable Skp2 knockdown genotype showed a less obvious spindle-type shape, as well as lower migratory and invasive capabilities. The sensitivity of resistant OS cells to methotrexate treatment was also improved by knockdown of Skp2 gene expression. These results suggested that depletion or silencing of Skp2 probably suppresses the EMT and restores the sensitivity of cancer cells to methotrexate and is therefore a potential therapeutic target [34].

The multiple factors involved in the resistance of cancer cells to methotrexate also participate in complex extracellular signal pathways, e.g., GOLM1 is a positive regulator of the PI3K/AKT pathway, and the PI3K inhibitor BKM120 abrogates the function of GOLM1 during the oncogenic process [35]. Skp2 has been reported to show crosstalk with several signaling pathways, such as the estrogen receptor pathway [36] and Notch signaling pathway [37]. Skp2 is also a component of the phosphatase and tensin homolog (PTEN)/PI3K pathway and is indispensable for the regulation of p27 and cell proliferation in carcinoma [38]. These observations suggest that the restoration of sensitivity to chemotherapeutic agents may be much more complicated than initially thought. On the other hand, accumulating knowledge about different cellular pathways also provides new insights to overcome drug resistance by utilizing combinations of drugs that target several receptors and pathways simultaneously.

### 2.2. 5-Fluorouracil

5-Fluorouracil (5-FU) is another antimetabolite used as the first-line chemotherapeutic agent in the treatment of various cancers. The primary target of 5-FU is the pyrimidine thymidine, which is required for replication of DNA. Disturbing the activity of thymidylate synthase leads to cell cycle arrest and apoptosis in cancer cells [39,40,41]. However, recent studies showed very low response rates (10–15%) to 5-FU in certain advanced carcinomas, such as chemoresistant colorectal cancer [42].

In humans, RNA binding protein, fox-1 homolog 3 (RBFOX3) is a protein-coding gene consisting of 15 exons located on chromosome 17 [43]. MicroRNAs (miRNAs)—short noncoding RNAs that regulate vital biological mechanisms, including differentiation, proliferation, and apoptosis [44,45,46]—are generated via two-step cleavage of primary microRNAs (pri-miRNAs) [47]. Previous studies showed that RBFOX3 can bind to these pri-miRNAs, and thus regulate microprocessor complex recruitment [48]. Liu et al. [49] also reported that RBFOX3 knockdown enhanced the sensitivity to 5-FU and inhibition of cell proliferation, migration, and invasion, both in vitro and in vivo, through targeting the PI3K/AKT pathway.

In another in vivo study [50], EMT-suppressive miRNAs were evaluated in two strains of 5-FU-resistant cells (HCT116-5-FUR and SW480-5-FUR; both human-derived colon cell lines) and susceptible parental cell lines. The levels of miR-34a, miR-200c, and miR-429 expression were markedly decreased in HCT116-5FUR and SW480-5FUR cells compared to the parental cell lines. Accompanying the variations in miRNAs, the level of ZEB1 protein was increased and E-cadherin was lost in these cells.

These observations indicated that the mesenchymal phenotype was gained during the development of 5-FU chemoresistance, suggesting that it may be possible to develop novel therapeutic strategies based on EMT-suppressive miRNAs, such as miR-34a, miR200c, and miR-429.

Zhang et al. [51] demonstrated that treatment of HCT1116-5FUR cells with a combination of curcumin and 5-FU upregulated the expression of EMT-suppressive miRNAs, such as miR-101, miR-141, miR-200b, miR-200c, and miR-429, thus improving drug sensitivity. They also reported that overexpression of miR-200c in HCT116-5FUR cells resulted in increased E-cadherin expression and downregulation of ZEB1, indicating suppression of the EMT.

A member of the zinc-finger E-box binding homeobox family, ZEB1, was deemed to be a key transcription factor in carcinogenesis [52]. ZEB1 binds to the promoter of E-cadherin and suppresses its transcription [53,54]. These observations demonstrated that ZEB1 plays a critical role in inducing and promoting EMT progression, therefore causing chemoresistance.

Similar to ZEB1, Snail has also been shown to play a major role in 5-FU-induced EMT in low- to advanced-grade malignant cancer cells [55]. Previous studies also showed that small interfering RNA (siRNA)-mediated knockdown of ZEB1 and Snail expression can markedly increase the sensitivity of cancer cells to chemotherapy [56].

The ATP-binding cassette (ABC) membrane transporter has been shown to participate in multidrug resistance [55]. During this process, 5′ AMP-activated protein kinase (AMPK) acts as an acellular energy sensor that, when activated, can increase the cellular AMP/ATP ratio, thus affecting drug resistance and inducing apoptosis of cancer cells [57].

Metformin, a well-known clinical activator of AMPK, was also found to induce HO-1 and the endogenous antioxidant, thioredoxin, thus inhibiting reactive oxygen species (ROS) production and suppressing the EMT induced by TGF-β pathway [58]. Other AMPK activators, such as AICAR, show the same effects as metformin. Increased expression of another target gene, HES1, was also demonstrated to accompany upregulation of ABC. Sun et al. [59] reported that higher levels of HES1 expression were associated with a higher recurrence rate and poorer prognosis after 5-FU-based chemotherapy in stage II colorectal cancer patients.

These observations suggest that further understanding EMT-suppressive miRNAs and the ABC family may lead to new breakthroughs for ameliorating chemoresistance to 5-FU.

### 2.3. Capecitabine

The use of capecitabine (Xeloda; N4-pentyloxycarbonyl-5′-deoxy-5-fluorocytidine) for treating HNC was first reported in 2002 [60]. Capecitabine is an oral fluoropyrimidine prodrug efficiently absorbed in the gastrointestinal tract; this is followed by a series of enzymatic conversions, with 5-FU being the final product in hepatic, extrahepatic, and malignant tissue [9]. Thus, both capecitabine and 5-FU have a largely identical molecular mechanism of action as first-line chemotherapeutic agents [61,62]. There is still some controversy regarding the clinical application of these two agents, although most authors agree that they can be used interchangeably (Table 2) [63].

It is noteworthy that capecitabine treatment induces approximately 2.9-fold higher 5-FU concentrations in malignant than nonmalignant tissue [64]. Moreover, oral administration offers the obvious advantage of convenience for patients in comparison to intravenous infusion of 5-FU [65]. In addition, capecitabine given orally demonstrates in a consistent higher ratio of tissue-to-plasma 5-FU concentration than 5-FU administered intravenously [66].

In preclinical evaluations, the antitumor and toxicity profiles of capecitabine were consistently superior to those of 5-FU [67]. Interestingly, previous exposure to cisplatin–5-FU combination chemotherapy did not seem to induce irreversible resistance to further cisplatin–capecitabine treatment. A similar observation was made in patients treated during a phase I trial of a combination of capecitabine and oxaliplatin [68]. The mechanism of drug resistance to capecitabine remains unclear. Research has mostly been concerned with 5-FU and been used to represent the drug resistance to capecitabine. However, further studies to determine whether capecitabine is truly pharmacologically identical to 5-FU are warranted.

**Table 2:** Comparison of 5-FU- and capecitabine-based chemotherapeutic treatments.

	Reference	Outcomes	5-FU	Capecitabine	*p*-Value
Administration			Intravenous administration	Oral administration	
Toxicity (Grade III/IV)	[69]	Diarrhea	12.7%	16.6%	0.001
	[70]	Diarrhea Stomatitis Hand-foot syndrome	58.2% 61.6% 6.2%	47.7% 24.3% 53.5%	<0.001 <0.001 <0.001
	[71]	Acute adverse effects	28.21%	26.82%	>0.05
	[72]	Stomatitis Hand-foot syndrome	16% 1%	3% 18%	<0.0001 <0.0001
Tumor Response	[73]	Overall response rate	17%	26%	<0.0002
	[71]	Complete pathological response rate	15.48%	19.53%	0.04
	[72]	Overall response rate	11.6%	25%	0.005
Survival	[74]	Overall survival (median survival in months)	12.1 M	13.2 M	0.33
	[75]	Overall survival (median survival in months)	12.8 M	12.9 M	0.05
	[71]	Three-year disease-free survival (rate)	75.72%	76.67%	0.05
	[72]	Overall survival (median survival in months)	13.3 M	12.5 M	0.05

According to the integrated analysis of Cassidy and Hoff et al. [70,72], capecitabine-based chemotherapy has a better safety profile than 5-FU-based regimens, with significantly lower incidence rates of grade III/IV chemotoxicity, such as diarrhea and stomatitis. However, the incidence of hand-foot syndrome was significantly higher with capecitabine-based treatment in comparison to 5-FU-based treatment, M: months.

## 3. Platinum-Based Agents

### 3.1. Cisplatin

Cisplatin is one of the most commonly used platinum-based chemotherapeutic agents and has shown efficacy in the treatment of various types of cancer, including breast cancer [76], lung cancer [77], and HNC [78,79]. The mode of utilization of cisplatin has undergone changes a number of times since it was first discovered in 1978 [80,81]. The development of cisplatin resistance by cancer cells has become an intractable problem worldwide.

Numerous strategies have been developed to increase the sensitivity of cancer cells to cisplatin treatment [82]. Propofol in combination with cisplatin was shown to suppress autophagy by downregulating the MALAT1/miR-30e/ATG5 axis and sensitizing gastric cancer cells to cisplatin [83].

Chemotherapy with cisplatin is associated with increased migration of classically activated macrophages, along with secretion of chemokine ligands 20 and 6 (CCL20 and CCL6, respectively) [84]. CCL20 recruits T cells to maintain the immunosuppressive environment and ensure cancer progression [85,86,87], while CCL6 can induce cancer cell migration and invasion [88]. The CCL20/CCL6 axis is activated by chemotherapy with cisplatin, leading to increased cancer cell migration and EMT-based resistance [84].

Cisplatin can increase EMT-AFTs to enhance the migration capability of tumor cells and reduce sensitivity to antitumor drugs. This cisplatin-induced EMT was considered to be unrelated to drug concentration and exposure period [89]. Ataxia telangiectasia mutated (ATM), a key member of the PI3K family, is involved in the DNA damage response and can be activated by endogenous and exogenous factors, such as ROS and irradiation, consequently triggering cell cycle checkpoint signaling, DNA repair, or apoptosis [90,91]. Another tumor suppressor factor, Schlafen 11 (SLFN11), was also suggested to participate in drug resistance/sensitivity, and both ATM upregulation and SLFN11 downregulation can activate EMT to stimulate resistance of tumor cells to cisplatin-based chemotherapy [92,93]. Some groups have also suggested that cisplatin causes EMT by activating the oncogenic NF-κB signaling pathway [94].

No single factor can induce complex drug resistance, but a number of diverse mechanisms are involved. Further understanding of the molecular signaling pathways underlying the resistance to antitumor drugs caused by EMT may facilitate the development of methods to increase the efficacy of chemotherapy treatment.

### 3.2. Carboplatin

Carboplatin is another platinum-based antineoplastic agent that works by interfering with DNA replication [95].

Both European and American guidelines recommend “platinum-doublet” regimens consisting of the combination of a platinum agent (cisplatin or carboplatin) with a second active drug for first-line treatment of metastatic non-small-cell lung cancer (NSCLC) [96,97,98]. Carboplatin has also been used to treat a number of cancers in addition to NSCLC, including HNSCC, brain cancer, and neuroblastoma [99].

A series of randomized clinical trials (RCTs) showed that such platinum-doublet chemotherapy can improve both survival and quality of life (QoL) compared to best supportive care alone.

Although both carboplatin and cisplatin are platinum-based chemotherapeutic agents, their toxicity profiles are different (Table 3) [100]. Therefore, the preferred combination in this setting is still a matter of some debate.

**Figure 2:**
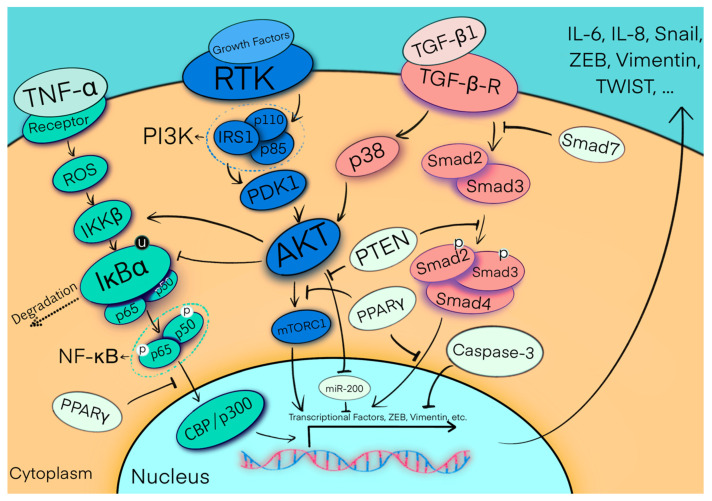
A diagram of the NF-κB (canonical), PI3K/AKT, and TGF-β pathways. NF-κB is shown in the figure as a heterodimer consisting of p65 and p50 subunits. In the canonical pathway, NF-κB is bound and inhibited by IκBα proteins. Binding of proinflammatory cytokines, such as TNF-α, to specific transmembrane receptors activates the IKKβ protein, which phosphorylates IκBα and leads to its ubiquitination and, finally, to proteasomal degradation. In this process, ROS interact with NF-κB in various ways, such as by activating IKKβ or IκBα phosphorylation. The active NF-κB complex translocates to the nucleus. For optimal activation of the transcription of NF-κB for certain target genes, interaction with CBP or p300 is required [137,138]. PI3K consists of two domains, p110 and p85, but its activation requires adapter molecules, such as IRS1. Following activation and phosphorylation of the tyrosine kinase receptor (TKR) by growth factors, TKR will recruit PI3K, finally activating AKT by recruiting phosphoinositide-dependent kinase-1 (PDK1) to the kinase domain of AKT. AKT can subsequently affect downstream factors and activate mTORC1, thereby activating the entire pathway and regulating cell growth and apoptosis. AKT can also be activated by p38, which is controlled by the TGF-β pathway. Activated AKT inhibits IκBα and miR-200, while triggering IKKβ protein to further affect cell immunity, apoptosis, and proliferation [139]. In the TGF-β pathway, members of the TGF-β superfamily, such as TGF-β1, bind to the transmembrane receptor (TGF-β-R), resulting in phosphorylation of the cytoplasmic signaling molecules Smad2 and Smad3. The activated Smad2/3 bind to the signaling transducer, Smad4, and this newly formed complex translocates to the nucleus [140]. There is a great deal of crosstalk between these signaling pathways, and the translocation process can also be inhibited by peroxisome proliferator-activated receptor-γ (PPAR-γ), PTEN, or caspase-3. However, once the functions of the suppressors are inhibited, transcription factors, such as ZEB, vimentin, etc., will be activated and secreted into the extracellular environment, eventually leading to conversion of the microenvironment. p: phosphorylation, u: ubiquitination.

Vanadium has been shown to be involved in several cellular pathways, and to possess antitumor potential [101]. A previous study showed that vanadium treatment upregulated E-cadherin and downregulated the expression of N-cadherin, which is a vital mesenchymal marker in human lung cancer, through the TGF-β pathway by activating Smad signaling [101]. Vanadium can suppress the expression of phospho-Smad2 and Smad2/3 nuclear translocation, which are induced by activation of the TGF-β pathway (Figure 2). The level of apoptosis of lung cancer cells was markedly increased by combined carboplatin and vanadium ex vivo treatment, but not by either agent alone [102]. With combination treatment, the percentage of cells in the G0/G1 phase was dramatically increased, while those in the S and G2/M phases were significantly decreased.

**Table 3:** Comparison of cisplatin- and carboplatin-based chemotherapeutic treatments.

	Reference	Outcomes	Cisplatin	Carboplatin	*p*-Value
Administration			Intravenous administration	Intravenous administration	
Toxicity (Grade III/IV)	[103]	Neutropenia Diarrhea Stomatitis	60.0% 1.9% 0.7%	51.5% 1.5% 0.0%	NC NC NC
	[104]	Nephrotoxicity Neurotoxicity	37% 33%	8% 4%	0.03 0.02
	[105]	Nausea/Vomiting Neutropenia	17.7% 0.0%	5.6% 1.1	0.0129 >0.05
Tumor Response	[103]	Objective response rate	49.3%	48.5%	>0.05
	[104]	Overall clinical response rate	71%	41%	0.04
	[106]	Objective response rate	33.75%	27.125%	0.001
	[107]	Objective response rate	57.03%	42.19%	0.02
	[105]	Objective response rate	35.6%	23.6%	0.05
Survival	[103]	Overall survival (median survival in months)	12.87 M	10 M	0.05
	[106]	Overall survival (median survival in months)	9.2 M	8.4 M	0.05
	[105]	Overall survival (median survival in months)	8.75 M	8 M	0.05

Previous comprehensive systematic reviews [103,104,105,106,107] indicated that treatment with these two platinum-based agents had similar therapeutic efficacy in terms of the survival rates but their safety profile and tumor response differed. NC: not compared in the article, M: months.

In addition to induction of the EMT by the TGF-β pathway, Notch is also considered to be a critical signaling pathway in the development of the EMT [108]. In mammals, the Notch family consists of four transmembrane receptors (Notch 1–4) and five ligands (Jagged1, Jagged2, and Delta-like ligands [Dll] 1, 3, and 4) [109]. The Notch pathway is activated when the cells come into contact with each other by binding of the Notch ligands onto the transmembrane receptors of neighboring cells. Once the Notch cascade has started, a series of proteolytic cleavages are initiated, including S3 cleavage mediated by the γ-secretase complex, leading to the release and nuclear translocation of the Notch intracellular domain (NICD) [110,111]. The NICD is able to convert the DNA binding protein CBF1/Su(H)/Lag-1 from a repressor to an activator of transcription, thus causing carcinogenesis [112,113]. Earlier studies also showed that overexpression of NICD3 (active form of Notch3) attenuates the expression of E-cadherin while increasing the expression of Snail, Slug, and smooth muscle α-actin (αSMA). Gupta et al. [109] reported that activation of Notch3 rendered cells more resistant to carboplatin-induced toxicity, based on their ex vivo study in which the human-derived ovarian cancer cell line OVCA429/NICD3 showed higher viability than control OVCA429/Vector cells on treatment with carboplatin. Furthermore, the carboplatin-induced cleavage of poly (ADP-ribose) polymerase (PARP) and caspase-3 was also reduced by activation of Notch3.

Taken together, these studies indicate that the combination of an inhibitor of TGF-β or Notch activation with carboplatin is more effective than either agent alone for attenuating tumor formation and metastasis.

## 4. Plant Alkaloids

### 4.1. Paclitaxel

Paclitaxel was first isolated from the bark of the Pacific yew as a chemotherapeutic agent in 1963 [114]. By targeting the microtubule cytoskeleton, paclitaxel interferes with mitotic spindle assembly, chromosome segregation, and cell division [115]. However, resistance to paclitaxel has become a common and intractable problem [116]. Paclitaxel resistance was suggested to be related to the acquisition of the mesenchymal phenotype by cancer cells during chemotherapy [117]. Park et al. [118] reported that once human breast cancer cells gain resistance to paclitaxel, they also transition to a more spindle-shaped morphology and the levels of the mesenchymal cell markers, vimentin and fibronectin, increased 2.5- and 1.5-fold, respectively.

The EMT is not only caused by chemotherapy with paclitaxel but is also induced by carcinoma-associated fibroblasts (CAFs) [119]. These CAFs induce the EMT of cancer cells via the IL-6/JAK2/STAT3 pathway by increasing the expression of the apoptosis-suppressing protein, Bcl-2, thus conferring apoptosis resistance and reducing the expression of the proapoptotic proteins, Bax and caspase-3 [120]. Finally, cancer cells develop EMT features leading to paclitaxel resistance.

Multivariate analysis showed that interstitial IL-6 expression was a critical and independent factor associated with paclitaxel resistance [121]. A retrospective study with a large sample size also revealed a similar outcome, in that patients with lower IL-6 expression showed a higher rate of sensitivity to chemotherapy than those with a higher level of IL-6 expression (69.3% and 48.1%, respectively) [122]. Although the reason is still unclear, Osuala et al. [123] confirmed that CAFs are the major source of IL-6 secretion. Based on this finding, suppression of interstitial IL-6 expression may reverse paclitaxel resistance.

Cathepsin L (CTSL) is a cysteine protease that has been reported to be associated with tumor development, recurrence, and metastasis [124,125,126]. Han et al. [127] developed a mouse model of CTSL overexpression; the expression of CTSL protein was significantly increased with paclitaxel treatment. Moreover, upregulation of CTSL enhanced the tolerance of breast cancer cells to paclitaxel in vivo. Zheng et al. [128] also reported that CTSL inhibition stabilized and enhanced the availability of cytoplasmic and nuclear protein drug targets. CTSL acts as an upstream regulator of NF-κB activation [129], and NF-κB has been shown to bind to the human Snail promoter in the region from −194 to −78 bp, and to upregulate its transcription [130].

As mentioned in the section on methotrexate, there is emerging evidence that Skp2 contributes to the resistance to paclitaxel [131]. Skp2 exerts its oncogenic functions via degradation of its ubiquitination targets, such as E-cadherin [132]. Moreover, high Skp2 expression level is associated with tumor recurrence [133], especially in tumor metastases to lymph nodes [134].

Variation of E-cadherin is associated with the migration capability of cells, with downregulation of E-cadherin enhancing mesenchymal traits in response to paclitaxel treatment [135]. TGF-β signaling was shown to be significantly increased based on detection of the phosphorylation of Smad2 and Smad3 in vivo and ex vivo when the cancer cells gained the mesenchymal phenotype after paclitaxel treatment (Figure 2) [136]. Notably, when Snail was inhibited by blocking TGF-β signaling, the sensitivity of cancer cells to paclitaxel also improved [137].

The evidence outlined above suggests that inhibiting NF-κB and TGF-β signaling, especially the activity of Snail, is vital for resensitization of cancer cells to paclitaxel therapy.

### 4.2. Docetaxel

Docetaxel is another representative taxane drug similar to paclitaxel. Their structures and mechanisms of action are largely the same, but they differ in several other aspects, such as tubulin polymer generation. In an ex vivo study, docetaxel also tended to be more potent in different cell lines; docetaxel is considered to be a schedule-independent drug, while paclitaxel is not [141,142]. Riou et al. [143] reported that docetaxel was 1.3–12-fold more effective than paclitaxel after 90 h of exposure, which may have been due to the higher affinity of docetaxel for microtubules.

Several factors have been shown to be associated with docetaxel resistance, including the expression of isoforms of β-tubulin, drug efflux pumps, and activation of survival factors (i.e., PI3K/AKT, mTORC) [144,145,146,147].

A gene expression analysis study showed that genes involved in the NF-κB pathway (NF-κB1, REL, RELA), androgen receptor (AR), and EMT-related genes (ZEB1, vimentin, and TGF-β-R3) were overexpressed in docetaxel-treated tumors [148]. The metastasis suppressor gene NDRG1 [149], adhesion molecule EPCAM [150], and negative regulator of ZEB1, ST14 [151], were shown to be decreased in treated tumors. Docetaxel-resistant prostate cancer cells show decreased expression of E-cadherin and CTNNB1 (epithelial markers), and increased expression of vimentin, TWIST1, and ZEB1. They also show stem-cell-like transcriptional features, including upregulated expression of CD44 and loss of CD24 [148].

Marín-Aguilera transfected ZEB1 siRNA into DU-145R and PC-3R cell lines (human-derived prostate cell lines) to interfere with the expression of ZEB1 [148], and reported that the transfected cells exhibited significantly increased sensitivity to docetaxel treatment. Moreover, the apoptosis induced by docetaxel was more pronounced in transfected cells, and the expression of CD44 was decreased after transfection. CD44 and CD147 can enhance metastatic capacity and chemoresistance, potentially via activation of the PI3K and MAPK pathways [152]. These observations indicated a link between EMT and a stem cell-like phenotype.

Treatment with docetaxel was also shown to upregulate the expression of cytokines, such as IL-6, via the NF-κB pathway [153]. Moreover, increased nuclear NF-κB was associated with a shorter time to clinical relapse [154]. The levels of miR-200c and miR-205, two miRNAs generally considered to regulate the epithelial phenotype, were also reduced in docetaxel-resistant prostate cancer cell lines.

## 5. Discussion

**Figure 3:**
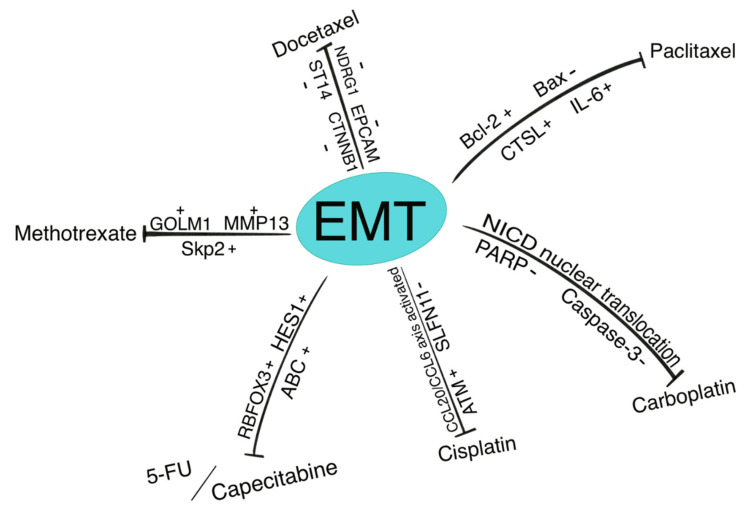
Variation of intra/extracellular factors during the EMT process. These expression changes underlie chemotherapeutic drug resistance. +: up-regulation, -: down-regulation.

In this review, we discussed the involvement of EMT in the development of resistance to several chemotherapeutic agents (Figure 3). These agents were listed by the NCCN as effective antitumor regimens that affect the viability of multiple types of tumor cells. Unfortunately, however, recent studies have demonstrated the potential of cancer cells, including OSCC cells, to develop resistance to various chemotherapeutic agents [10,11]. The EMT process is one of the most important mechanisms associated with resistance to chemotherapy.

Currently, the most common methods of overcoming such resistance in clinical settings involve the application of antitumor drugs along with chemotherapeutic agents [51,107]. The development of an effective regimen for activation of chemosensitivity relies on determining the complex molecular signaling pathways and mechanisms by which tumor cells develop drug resistance. Extensive studies of EMT and chemotherapy have explored numerous diverse targets for chemoresistance/chemosensitivity [12,19].

Many stimuli, including TGF-β, Shh, Wnt, inflammatory cytokines, and hypoxia, as well as oncogenes such as ErbB2 and mutant p53, may participate in EMT during carcinogenesis [155,156]. In addition to these stimuli, triggering and maintaining the EMT process requires cooperation between several pathways via autocrine signaling loops (Figure 2) [155].

Deregulation of E-cadherin is a vital initial event in the EMT. On the one hand, a decline of E-cadherin disrupts the close junctions between epithelial cells, leading to migration and invasion of the cells. On the other hand, loss of E-cadherin reinforces the EMT via upregulated expression of Snail, TWIST, ZEB1, etc. Downregulation of E-cadherin and upregulation of those ATFs result in a canonical feedforward loop (Figure 1) [157]. In this feedforward loop, the primary tumor cells secrete various cytokines and proteases, which help the tumor cells to create a peritumoral extracellular environment [158]. In turn, the stromal cells are also activated to release factors that strengthen the EMT in primary tumor cells, and foster cell survival and evasion of the immune system, which influences the tumor microenvironment [159,160]. EMT is necessary for tumor cells, because invasion requires the mesenchymal phenotype and a lack of tight junctions. A great deal of research has focused on the TGF-β signaling pathway, as it is assumed to play an important role in the transformation from the epithelial to mesenchymal cell phenotype [161,162].

In addition to gaining mesenchymal characteristics, cancer cells are also endowed with some stem-cell-like features during the EMT process via gene reprogramming [163,164]. Therefore, in addition to the TGF-β pathway, many other signals that participate in normal stem cell homeostasis are also involved in the EMT and seem to induce the generation and maintenance of CSCs.

The EMT-ATFs, including members of the Snail family, play important roles in repressing the expression of E-cadherin. The Snail family members Snail1, Snail2, and Snail3 can all repress genes involved in conferring epithelial characteristics through interactions with Smad2/3/Smad4 [165]. The binding of AKT1 to the E-cadherin promoter is decreased by Snail overexpression, triggering the activation of AKT2, a repressor that has the opposite effect to AKT1 [166]. The Snail proteins are induced by several pathways, including the TGF-β [167], Notch [168], TNF-α [169], hypoxia [170], and Wnt pathways [171].

TWIST is another very important family of transcription factors; this family includes TWIST1 and TWIST2, which share a basic/helix-loop-helix domain [172]. The binding of TWIST to E-boxes in the promoter regions of target genes, such as N-cadherin and AKT2, results in activation of their expression and inhibition of E-cadherin expression [173]. The TWIST factors, as well as inflammatory cytokine receptors, are upregulated by the activated EMT [174,175]. EGF and IL-6 induce TWIST1 via activation of the JAK/STAT signaling pathway and form a negative feedback loop with TWIST1 and TWIST2 [176,177,178].

The ZEB family, comprised of ZEB1 and ZEB2, can trigger EMT by repressing epithelial biomarkers and activating mesenchymal biomarkers [179,180]. Although ZEB1 and ZEB2 are distinct from each other, they have overlapping effects [181]. Activation of the TGF-β pathway induces both ZEB proteins, which in turn modulate the TGF-β pathway in different ways; ZEB1 acts synergistically with receptor-regulated Smads, while ZEB2 has a suppressive effect [179,181].

Many studies have focused on the roles of miRNAs in each step of cancer progression. A number of miRNAs have been shown to interact with the transcripts of numerous target genes involved in the EMT process, therefore regulating the migration and invasiveness of cancer cells [182]. Studies have demonstrated the interactions between EMT-AFTs and several miRNAs, including miR-218, miR-200b, miR-146b, miR-338-3p, miR-363, miR-139-5p, etc. [183,184]. ZEB suppresses miR-183, miR-203, and miR-205, which have similar functions to miR-200, deregulating the expression of stemness factors such as Bmi1 and Sox2 [185,186]. The miR-9, was also shown to downregulate the expression of Snail, thus inhibiting the proliferation and invasion of melanoma cells [187]. Snail1/2 and ZEB1 transcriptionally repress miR-34a/b/c to form a double-negative feedback loop, further promoting the expression of stem cell-related factors such as Bmi1, CD44, and CD133 [188]. The miR-200 family (including miR-200a/b/c, miR-141, miR-429, etc.) maintain the epithelial characteristics and prevent EMT by suppressing ZEB1, as well as Snail and TWIST [189,190,191]. Interestingly, only a few miRNAs were shown to be related to the TWIST factors. The miRNAs miR-29b, miR-214, and miR-580 were shown to inhibit the expression of TWIST; however, it is not clear whether the downregulation of TWIST1 was caused by the inhibition of Snail [192]. On the other hand, TWIST1 was shown to indirectly affect the expression of miR-10b, miR-199a, and miR-214 by binding to E-boxes in their promoters [193,194].

The manipulation of miRNA expression may represent a means of inhibiting EMT-mediated resistance to chemotherapy agents. Compared to the permanent impact of mutations and deletions, the expression of miRNAs and EMT-ATFs can be dynamically regulated, making them attractive targets for personalized oncological treatment [195]. As chemotherapy usually targets a single oncogenic signal and is therefore inevitably associated with the development of resistance or recurrence, multiple simultaneous approaches to various pathways and cancer cell traits may yield better results [196]. It is necessary to gain a better understanding of the EMT and its ATFs, along with the accumulation of more information about their upstream regulatory networks and mechanisms of action.

Finally, it should be noted that most studies performed to date were based on in vitro and in vivo experiments; there is a paucity of data from clinical trials. Therefore, a great deal of work is still necessary to establish reliable strategies for inhibiting the EMT, and the mechanisms by which it induces resistance to chemotherapy. Nevertheless, the results reported to date suggest the potential of studies of the EMT process to facilitate the development of novel and precisely targeted therapeutic approaches to cancer.

## 6. Future Perspectives

Extensive studies have provided a comprehensive understanding of the molecular pathways and ATFs involved in the EMT of OSCC/HNSCC. These insights have suggested the potential benefits of anti-EMT therapies.

Metformin, vanadium, etc., were shown to suppress markers of mesenchymal differentiation, such as vimentin and N-cadherin, while inducing the expression of E-cadherin. Previous in vivo studies showed that combining chemotherapy agents improved drug sensitivity and reduced the expression of E-cadherin, thus suppressing the EMT [51,58,107].

High-throughput screening systems have also been developed for identifying anti-EMT drugs. In a pilot screen using a novel three-dimensional high-throughput screening system for a test of 1330 compounds, Arai et al. [197] identified nine compounds that were above the thresholds and two of those compounds, the TGF-β-R1 inhibitor SB-525334 and CDK2 inhibitor SU9516, acted as inhibitors of EMT in lung cancer cell lines. Similarly, Germain et al. [198] identified a chemical probe, ML245, through high-throughput screening that restrained CSCs progression by regulating the expression of proapoptotic/mitochondrial maintenance factors and DNA-modifying enzymes.

Recent studies have focused on RNA interference by miRNAs [199]. Many have proposed their use as new therapeutic agents due to their ability to interfere with the EMT and downregulate specific regulators of mesenchymal differentiation. For example, miR-186 targeting of the 3′-UTR of Skp2 led to reduced Skp2 expression, p27 upregulation and cell proliferation [200]. Furthermore, miRNAs implicated in lipid metabolism (i.e., miR-10 and miR-130 a/b) modulate PPAR-γ expression; thus, this class of miRNAs could improve drug efficacy and safety [201,202,203]. Exogenous expression of miRNA-203 in colorectal cancer promotes chemoresistance by targeting ATM [204]; however, overexpression of miR-1915 sensitized these cells to multiple drugs by targeting the 3′-UTR of Bcl-2 mRNA [205]. While miRNA-based treatments show high promise, they are limited due the need for precise delivery systems and control of the immune response [206].

Unlike sarcomas and hematopoietic malignancies, OSCCs are characterized by high tumor mutational burden (TMB) and are often recognized as “nonself” by T-cells [207]. To evade immunodetection, epithelial cells must undergo phenotypic changes, likely through the EMT process. Thus, a deep understanding of the EMT mechanism is important for effective OSCC drug design. Hodges et al. [208] reported a higher frequency of STK11 or KEAP1 mutations in epithelial versus mesenchymal lung adenocarcinomas, implying that mesenchymal tumors have fewer neoantigen mutations; this may limit T-cell recognition and therapeutic efficacy. On the other hand, a positive correlation between TMB and TGF-β pathway activation, a predominant driver of the EMT, has been reported [209,210,211]. We suspect that the EMT plays a fundamental role in altering the tumor microenvironment, especially in high TMB cancers like OSCC. We hypothesize that OSCC patients will benefit from immune checkpoint blockade therapy. Previous studies have relied on static measurements of biomarkers of the EMT process. This approach does not completely reflect reality since the EMT is dynamic and reversible [212]. Progressive changes to gene and protein expression may also promote the mesenchymal-epithelial transition (MET) and subsequent tumor growth and spread. Support for this hypothesis comes from the observed downregulation of E-cadherin expression during carcinogenesis [213]. While the precise role of the MET in cancer progression is unclear, it does complicate the interpretation of classic EMT biomarkers, such as E-cadherin, Snail, and vimentin. Overall, improved methods are needed to understand the dynamics of gene and protein expression in this context. Serial tumor biopsies and identification of new EMT-specific biomarkers not expressed by stroma cells would address these limitations.

A great deal of progress has been made in understanding the EMT over the last several decades. It is anticipated that combined administration of antitumor drugs with chemotherapeutic agents, together with the development of innovative high-throughput screening and miRNA technology to acquire specific inhibitors targeting multiple pathways and ATFs, will translate into new clinical treatments for cancer, including OSCC/HNSCC, in the near future.

## 7. Conclusions

The EMT can promote resistance of cancer cells to a range of chemotherapeutic agents. Several signaling pathways and EMT-AFTs have been shown to play vital roles in this process. Further extensive studies of the complex pathways involved in the EMT and drug resistance, combined with innovative techniques such as high-throughput screening and miRNA-based technologies, will facilitate the development of precise strategies for the treatment of OSCC/HNSCC and other types of cancer.

## Figures and Tables

**Table 1:** Classification of various chemotherapeutic agents.

Categories	Agents
Antimetabolites	Methotrexate
	5-Fluorouracil
	Capecitabine
Platinum-based agents	Cisplatin
	Carboplatin
Plant alkaloids	Paclitaxel
	Docetaxel

## Data Availability

All data have been illustrated in the manuscript.
